# Topical CpG Oligodeoxynucleotide Adjuvant Enhances the Adaptive Immune Response against Influenza A Infections

**DOI:** 10.3389/fimmu.2016.00284

**Published:** 2016-07-29

**Authors:** Wing Ki Cheng, Adam William Plumb, Jacqueline Cheuk-Yan Lai, Ninan Abraham, Jan Peter Dutz

**Affiliations:** ^1^Department of Dermatology and Skin Science, Faculty of Medicine, Child and Family Research Institute, The University of British Columbia, Vancouver, BC, Canada; ^2^Department of Microbiology and Immunology, Faculty of Science, Life Sciences Institute, The University of British Columbia, Vancouver, BC, Canada; ^3^Department of Zoology, Faculty of Science, Life Sciences Institute, The University of British Columbia, Vancouver, BC, Canada

**Keywords:** influenza vaccines, CpG oligodeoxynucleotide, skin vaccination, T cell memory, antibody production

## Abstract

Current influenza vaccines generate humoral immunity, targeting highly variable epitopes and thus fail to achieve long-term protection. T cells recognize and respond to several highly conserved epitopes across influenza serotypes. A strategy of raising strong cytotoxic T cell memory responses to epitopes conserved across serotypes would provide cross serotype protection, eliminating the need for annual vaccination. We explored the adjuvant potential of epicutaneous (ec) and subcutaneous (sc) delivery of CpG oligodeoxynucleotide in conjunction with sc protein immunization to improve protection against influenza A virus (IAV) infections using a mouse model. We found enhanced long-term protection with epicutaneous CpG ODN (ecCpG) compared to subcutaneous CpG ODN (scCpG) as demonstrated by reduced viral titers in the lungs. This correlated with increased antigen-specific CD8 T cells in the airways and the lungs. The memory T cell response after immunization with ecCpG adjuvant was comparable to memory response by priming with IAV infection in the lungs. In addition, ecCpG was more efficient than scCpG in inducing the generation of IFN-γ producing CD4 T cells. The adjuvant effect of ecCpG was accompanied with its ability to modulate tissue-homing molecules on T cells that may direct them to the site of infection. Together, this work provides evidence for using ecCpG to induce strong antibody and memory T cell responses to confer protection against IAV infection.

## Introduction

Influenza virus belongs to the *Orthomyxoviridae* family of enveloped negative-sense, single-stranded segmented RNA viruses. Of the three subtypes of influenza viruses, influenza A virus (IAV) can infect many different species, including humans, other mammals, and birds. IAV is a highly contagious human respiratory pathogen and the cause of all influenza pandemics with a large impact on global health. Annual vaccination against seasonal influenza epidemics is recommended by governmental health organizations (1).

Current inactivated influenza vaccines generate a strong antibody response that is moderately protective against the targeted IAV strains (2). However, they do not generate heterotypic immunity that would be protective against a wide range of IAV strains, and only protect against the strains in the vaccine. Although current IAV vaccines can induce a strong humoral immune response, this response targets highly variable and rapidly changing epitopes on influenza hemagglutinin and neuraminidase (1). Thus, vaccination may offer little protection if the predominant IAV strains for the upcoming year are not well matched to the strains used in the vaccine (3). Furthermore, protection will wane over time as the prevailing IAV strains undergo genetic drift in the epitopes targeted by the vaccine (4). During the most recent IAV pandemic in 2009, the swine H1N1 strain infected an estimated 24% of the world’s population and was responsible for nearly 300,000 deaths (5, 6). The variable effectiveness of the seasonal IAV vaccines and the need to be immunized every year demonstrates the need for a universal IAV vaccine.

Although antibodies from B cells prevent the infection of cells by viruses, T cells are essential to eliminate infected cells. Cytotoxic CD8 T cells (CTLs) are responsible for the elimination of most IAV infected cells (7). Mice lacking CD8 T cells have a much higher mortality rate (8). T cells recognize highly conserved IAV epitopes; in humans, T cells respond to epitopes within the IAV proteins, M1 and nucleoprotein (9–11). These epitopes undergo little genetic drift and are highly conserved across IAV strains (12, 13). Indeed, CD8 T cells specific to conserved viral epitopes were protective against symptomatic H1N1 influenza in the absence of cross-reactive neutralizing antibodies (14). The low variability of influenza epitopes for cross-serotype T cell protection makes generation of a strong memory T cell response an attractive option for making a universal IAV vaccine. However, unlike neutralizing antibodies, memory T cells alone cannot completely block IAV infection (15). Thus, an ideal universal IAV vaccine should be capable of generating both a strong neutralizing antibody and a long-lived memory T cell response.

Vaccine efficacy is highly dependent on the route of delivery and its ability to properly stimulate the immune system (16). Optimizing the route of delivery and choice of adjuvant are essential for generating optimal quality and strength of the immune response. Adjuvants can be utilized to induce the desired type of immune response to a vaccine for protection. The two adjuvants currently approved in licensed vaccines in the United States are aluminum hydroxide (alum) and monophosphoryl lipid A. The mode of action of alum is not well understood but it appears to be independent of pattern recognition receptor signaling. Alum preferentially generates Th2-biased immune responses, while monophosphoryl lipid A is a TLR4 agonist that induces Th1-type immune responses (17–19). Finally, topical application of TLR7 agonists at the time of influenza vaccination has been shown to improve antibody responses to influenza in the elderly (20).

The TLR9 agonist CpG oligodeoxynucleotide (ODN), mimics DNA patterns that are common in bacteria but rarely found in humans (21, 22). It has recently shown promising results as a novel adjuvant to boost immune responses and is currently in clinical trials as an adjuvant and immune modulator (23–25). Adjuvant CpG ODN enhances immune responses when co-administered with protein antigens. This was demonstrated for several clinically relevant pathogens, including Hepatitis B and anthrax (23–25). Importantly, CpG ODN stimulated both strong Th1 and B cell responses, which was similar to the immune response to IAV (26). CpG ODNs have recently been studied as IAV vaccine adjuvant in chicken (27), ducks (28), and murine models (29) delivered via the intramuscular, intradermal, and intranasal routes, respectively.

Delivering a vaccine at the site of natural infection is often effective at generating protective immunity. However, intranasal delivery of adjuvanted vaccine in a mouse model can result in the generation of detrimental Th17 cells (30). Thus, research into novel delivery routes to generate a safe, protective anti-IAV T cell response is warranted. Skin delivery of vaccines is a promising approach to generate protective immunity in many vaccination settings. Interestingly, although a primary immune response is initiated in the skin after viral skin infection, migratory and resident memory T cell populations are observed at diverse mucosal sites (31). These memory T cells are long-lived and are able to protect against reinfection at distal areas of the skin. Transcutaneous immunization against *Chlamydia muridarum* and *Schistosoma mansoni* elicited protective responses when challenged intravaginally or in the airways, respectively (32, 33). Importantly, these protective responses involve both humoral and cell-mediated contributions (34, 35).

The ability of adjuvant CpG ODN to help induce protective immune responses to vaccination through the skin is an active area of research. Addition of CpG ODN to *Leishmania major* vaccination induced strong and protective Th1 and Th17 responses (36). Topical adjuvant CpG ODN generated a robust antigen-specific CTL response when administered with the OVA protein model antigen (37). Enhanced specific immune responses can protect against subsequent *Listeria monocytogenes* infection (38). Importantly, the route of application was critical for CpG ODN to optimally boost T cell responses. While intramuscular and subcutaneous administration induced antigen-specific CD8 T cells, administration of CpG ODN by the transcutaneous route resulted in an enhanced CD8 T cell response that mediated protection.

As topical adjuvants can increase vaccine efficacy and promote T cell responses to vaccine antigen, we sought to harness immune cells in the skin to induce effective, long-lasting cellular immune responses, to potentially protect against different influenza serotypes. We investigated whether CpG ODN applied topically to the skin at the time of subcutaneous protein vaccination could help drive a protective T cell response against IAV. We found that epicutaneous CpG ODN (ecCpG) drove both CD8 T cell and antibody responses against IAV. These responses were sufficient to protect against subsequent IAV challenge in the lungs. Our work demonstrates the potential efficacy of immunization against IAV with adjuvant CpG ODN in the skin to drive a cross-protective T cell response that maybe relevant in influenza vaccine design.

## Materials and Methods

### Mice

C57BL/6 mice were purchased from Charles River Laboratories (Wilmington, MA, USA). The B6.Cg-Tg (TcraTcrb) 425cbn/J mice expressing a T cell receptor specific to OVA_323–339_ in the context of I-A^b^ (OT-II transgenic mice) and the B6.PL-Thy1^a^ mice that carry the congenic Thy1.1 allele (Thy1.1 mice) were from The Jackson Laboratory (Bar Harbor, ME, USA). The Thy1.1 and the OT-II mice were crossed to generate Thy1.1^+/+^ OT-II^+/+^ mice. All mice were housed in a specific pathogen-free condition at the Child and Family Research Institute (Vancouver, BC, Canada) or at the Centre for Disease Modeling (Vancouver, Canada). Animal experiments were conducted in accordance with the protocols approved by the University Animal Care Committee and Canadian Council of Animal Care guidelines. Age-matched female mice were used between 6 and 12 weeks of age.

### Viruses

The influenza strains used in this study are H1N1 A/Puerto Rico/8/34 (PR8) and H3N2 A/X-31, A/Aichi/68 (X31), and their derivatives PR8-OVA, and X31-OVA. PR8 is a mouse-adapted human IAV strain, while X31 is derived from the PR8 strain with its hemagglutinin and neuraminidase genes replaced by those from H3N2 A/Hong Kong/1/1968 (39). The PR8-OVA and X31-OVA strains contain the CD8 T cell epitope SIINFEKL (OVA_257–264_) in the neuraminidase stalk (40). PR8 and X31 were purchased from Charles River Laboratories (Wilmington, MA, USA). PR8-OVA and X31-OVA were grown in-house as previously described (41).

### Deoxynucleotides

Synthetic HPLC-purified, single-stranded, phosphorothiolated CpG ODN 1826 (5′-TCCATGACGTTCCTGACGTT-3′) was purchased from Sigma-Aldrich Inc. (Saint Louis, MO, USA). Lyophilized CpG ODN 1826 was reconstituted to 5 mg/mL with PBS/DMSO (1:1 v/v).

### Immunization

Mice were immunized as previously described (42). Mice were anesthetized by intraperitoneal injection of 75 mg/kg ketamine (ketalean, Bimeda-MTC Animal Health Inc., Cambridge, ON, Canada) and 7.5 mg/kg xylazine (Rompun, Bayer Health Care Inc., Toronto, ON, USA). The backs of the mice were then shaved, tape-stripped 15 times using cellophane tape (Staples, Vancouver, BC, Canada). The exposed skin was gently rubbed with acetone (Fisher Scientific, Edmonton, AB, Canada) using a cotton swab. One hundred micrograms of chicken ovalbumin antigen (OVA, Grade V, Sigma) in PBS were injected subcutaneously. CpG ODN in PBS/DMSO as adjuvant was administered either epicutaneously (250 μg, epifocal to the antigen injection site on a 1 cm × 1 cm 1-ply paper towel) or co-injected with OVA subcutaneously (50 μg). For experiments examining the IAV antibody response, PR8 was heat-inactivated for 2 h at 56^o^C before administered with CpG ODN as above. The immunization site was protected with water-resistant tape (Transpore 3 M) overnight. For a prime-boost immunization schedule, mice were immunized twice, 7 days apart.

### Influenza A Infection

For immunizing infection, mice were anesthetized using isoflurane and infected twice intranasally with 10 hemagglutinin units (HAU) of X31 or 20 HAU of X31-OVA in 25 μL of sterile PBS, 7 days apart. To challenge the immunized mice, mice were infected intranasally with 5 HAU of PR8 or 10 HAU of PR8-OVA in 25 μL of sterile PBS at 5 or 30 days after the booster or primary infection.

### Plaque Assays

Plaque assays were performed as previously described (43). Three days after infection with PR8-OVA lungs from infected mice were homogenized using a Fisher Tissuemiser, diluted in PBS and incubated on at least 80% confluent MDCK cells (ATCC) for 1 h at room temperature (RT). The wells were rinsed with PBS and a solution of 0.7% agarose, 0.1% trypsin in α-MEM was applied and allowed to solidify. Samples were then incubated at 37°C for 4 days before staining with crystal violet and counting of plaques.

### Adoptive Transfer of T Cells

Single cell suspension was prepared from lymph nodes (cervical, axillary, brachial, pancreatic, inguinal, and mesenteric) and the spleen of Thy1.1 OT-II mice by forcing through a 40 μm filter. Single cell suspension was enriched for CD4 T cells (>85% purity) by negative selection using EasySep^™^ Mouse CD4 T Cell Enrichment Kit (STEMCELL Technologies Inc., Vancouver, BC, Canada). 2 × 10^6^ cells were adoptively transferred via lateral tail vein injection 1 day before the experiment.

### Cell Preparation and Flow Cytometry

Broncheoalveolar lavage (BAL) fluid was obtained by inserting a tracheal catheter and washing the bronchoalveolar space four times with 1 mL of PBS supplemented with 10% FBS. Lymphocytes were extracted from the lungs of mice by mincing with scissors, digesting with 100 units/mL collagenase IV for 1 h at 37°C before filtering through a 70 μm filter to remove debris. Single cell suspension of spleens and skin draining lymph nodes (axillary and brachial) were prepared as described above. Erythrocytes in spleen were lysed with ACK lysis buffer before proceeding to staining.

Antibodies were purchased from BD Bioscience (San Diego, CA, USA), eBioscience (San Diego, CA, USA) and BioLegend (San Diego, CA, USA). H2-K^b^ OVA_257–264_ tetramer labeled with PE was made in-house by Dr. Rusung Tan’s Laboratory (Child and Family Research Institute, Vancouver, BC, Canada). H2-K^b^ NP_366–374_ and PA_224–233_ tetramers labeled with PE and APC, respectively, were made by the NIH Tetramer Core Facility (Atlanta, GA, USA). Purified mouse P-selectin–Ig fusion protein was purchased from BD Bioscience (San Diego, CA, USA) and the recombinant mouse E-selectin Fc chimera was from R & D Systems Inc. (Minneapolis, MN, USA).

Tetramer staining was carried out at RT for 30 min before antibody staining at 4°C for 30 min. For intracellular IFN-γ staining, cells were restimulated with PMA and ionomycin *ex vivo* at 37°C for 4 h before surface markers staining at 4°C for 30 min. Cells were fixed and permeabilized with Fixation and Permeabilization buffer from eBioscience and then stained for intracellular IFN-γ at RT for 30 min. Data were acquired on a LSRII flow cytometer using FACSDiva software (BD Bioscience) and analyzed using FlowJo software (TreeStar, Stanford, CA, USA).

### Detection of Antigen-Specific Antibodies by ELISA

Nunc 96-well Flat Bottom Immuno Plates were coated with 2 μg of OVA protein (Sigma) in 100 μL of 50 mM bicarbonate/carbonate buffer, pH 9.6 buffer, or 500 HAU/mL of inactivated PR8 per well. For OVA-specific antibodies, plates were blocked with 2% bovine serum albumin (BSA) in PBS at 37°C for 1 h. Twofold serial dilutions of sera were incubated on the plates at RT for 2 h and bound antibodies were detected with horseradish peroxidase (HRP)-linked Goat Anti-Mouse IgG and IgG2c antibody from Jackson ImmunoResearch (West Grove, PA, USA). Plates were washed between each step with 0.05% Tween in PBS. Tetramethylbenzidine substrate was added for signal detection and the reaction was stopped with 2N sulfuric acid. Absorbance was measured at 450 nm. For influenza-specific antibodies, plates coated overnight at 4°C with heat-inactivated IAV and were blocked with 1% BSA and 0.1% Tween in PBS at RT for 1 h. Fivefold serial dilutions of sera were incubated on the plates at 4°C overnight and bound antibodies were detected with biotin-conjugated anti-mouse IgG2b at 37°C for 2 h and avidin-HRP in blocking buffer at RT for 45 min. Substrate 2,2′-azino-bis(3-ethylbenzothiazoline-6-sulphoic acid) was added and the reaction was stopped with 2N sulfuric acid. Absorbance was measured at 490 nm.

### Statistical Analysis

Data are presented as mean ± SEM and analyzed by Student’s *t*-test or one-way ANOVA with Tukey’s post-test as appropriate. Results giving a *p*-value of less than 0.05 were considered significant.

## Results

### CpG ODN Adjuvant Enhances Protection from Influenza A Virus Challenge

**Figure 1:**
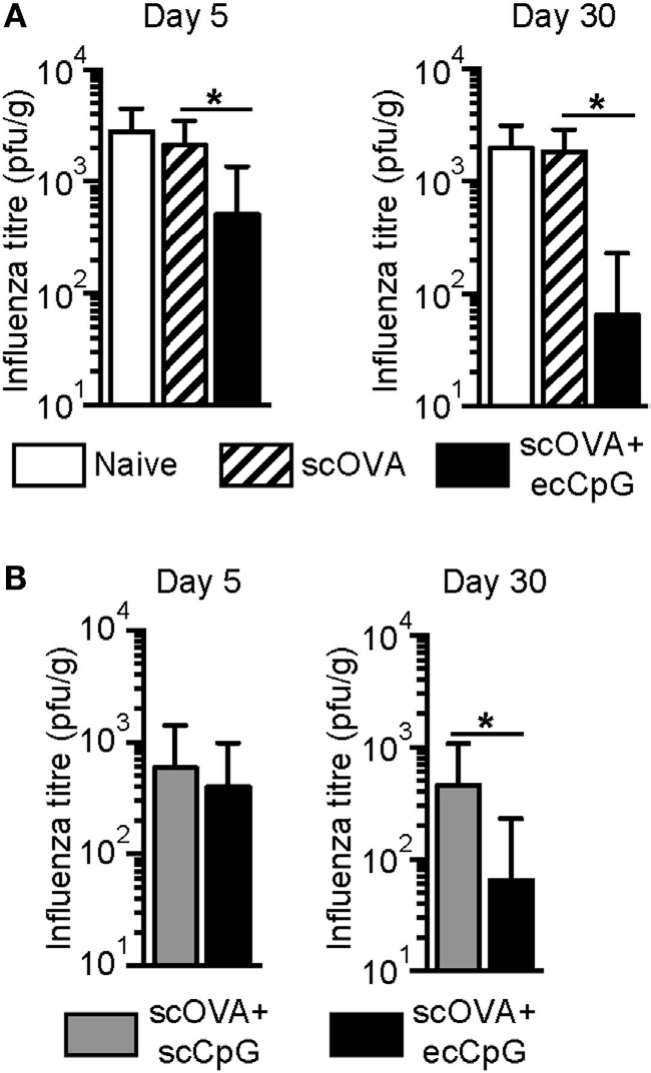
**Epicutaneous CpG ODN adjuvantation at the time of immunization protects against flu infection**. C57BL/6 mice immunized with **(A)** scOVA ± ecCpG ODN or **(B)** scOVA + sc or ecCpG ODN were infected with PR8-OVA virus intranasally 5 or 30 days post-immunization. Three days post-infection, lung homogenates were analyzed for plaque forming units. Bar graphs represent mean ± SD from two independent experiments (*n* = 9–11). Statistical significance comparing the immunized groups was analyzed by non-parametric two-tailed Mann–Whitney test *p < 0.05.

To assess the ability of ecCpG adjuvant to provide protection from intranasal IAV infection, naive C57BL/6 mice or mice immunized with the OVA antigen subcutaneously (scOVA) or scOVA with ecCpG adjuvant were challenged with PR8-OVA 5 or 30 days post-immunization. At 3 days post-infection, lung homogenates from challenged mice were analyzed by plaque assay to assess viral loads. The number of plaques was significantly lower in the ecCpG adjuvanted mice compared to mice given scOVA alone. This difference was seen in mice that were challenged 5 or 30 days post-immunization (Figure 1A).

Since ecCpG adjuvant was superior to subcutaneous CpG ODN (scCpG) adjuvant in providing protective immunity to *L. monocytogenes* (38), we further assessed whether ecCpG adjuvant is superior to scCpG adjuvant in providing protection from IAV. Mice were immunized with either scOVA and scCpG, or scOVA and ecCpG. Mice were challenged with PR8-OVA either 5 or 30 days post-immunization and lungs were harvested for plaque assays 3 days later. Interestingly, significantly lower number of plaques was detected in the lung homogenates from mice given CpG adjuvant epicutaneously compared to subcutaneously at day 30 post-infection (Figure 1B). These results show that administering CpG ODN adjuvant epicutaneously reduces viral load in the lungs, which suggests a protective effect of the immunization. In addition, ecCpG compared to scCpG adjuvant is more effective after 30 days in enhancing protection against IAV challenge.

### Epicutaneous CpG ODN Adjuvant Drives Memory CD8 T Cell Response in the Lungs

**Figure 2:**
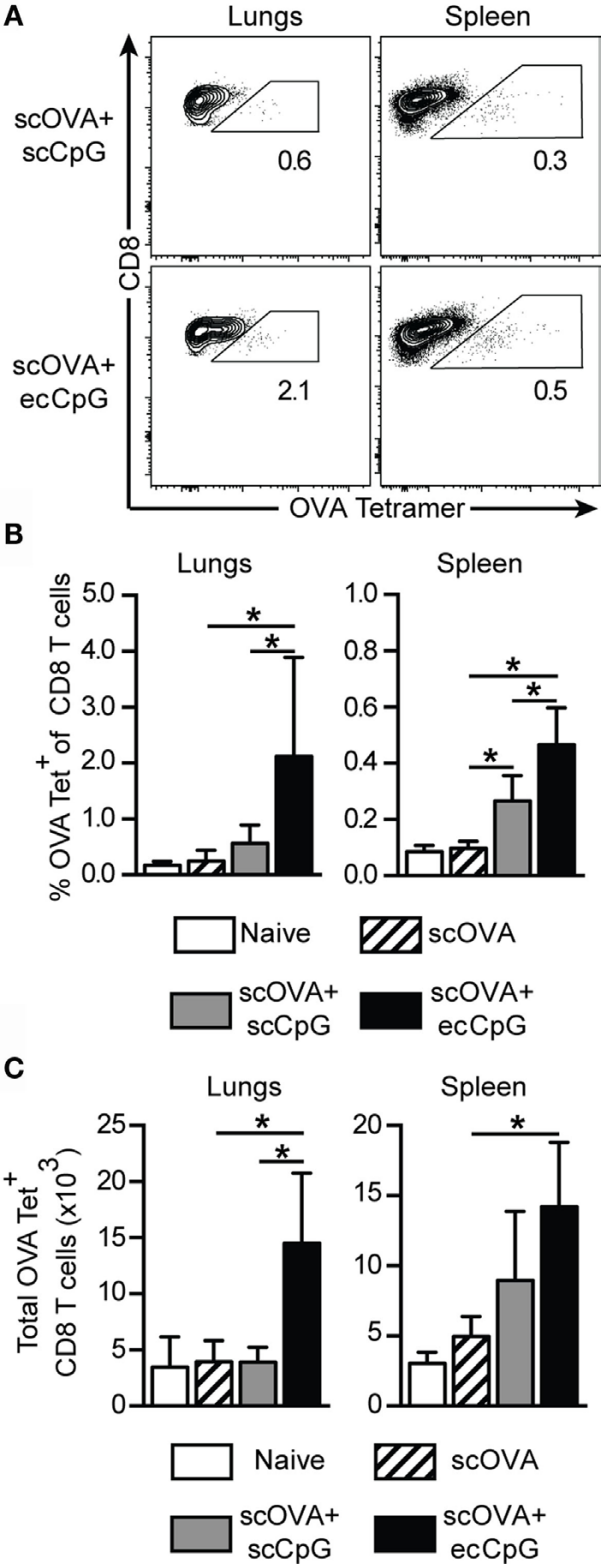
**Epicutaneous CpG ODN induces long-term CTL memory**. C57BL/6 mice immunized with scOVA ± ecCpG or scCpG ODN were restimulated with scOVA antigen at the same site of immunization 100 days post-immunization. Lungs and spleen were collected for flow cytometric analysis and OVA-specific CD8 T cells were enumerated. **(A)** Representative flow cytometric plots of the B220^−^CD8^+^ population, gating for OVA tetramer and CD8 double-positive cells. **(B)** The percentage and **(C)** total number of OVA-specific tetramer^+^ cells among CD8^+^ T cells in the lungs and spleen. Bar graphs show the mean ± SD from two independent experiments (*n* = 4–6). Statistical significance of all four groups was analyzed by one-way ANOVA test with a Tukey’s multiple comparison *post hoc* analysis *p < 0.05.

Memory CD8 T cells recognize conserved proteins, including nucleoprotein from different subtypes of IAV and, thus, may be harnessed to broaden protection against the virus (44). We speculated that memory CD8 T cells contributed to the protection from IAV infection driven by scOVA + ecCpG immunization. Therefore, we investigated the ability of ecCpG adjuvant to drive the memory CD8 T cell response. Mice were restimulated 100 days after immunization with scOVA at the site of primary immunization and the proportions of OVA-specific CD8 T cells in different sites were analyzed by flow cytometry. The total number of OVA-specific CD8 T cells in the lungs and the spleen were also enumerated. Immunization with CpG ODN adjuvant increased the percentage of OVA-specific CD8 T cells in the spleen and the lungs (Figures 2A,B). This increase in OVA-specific CD8 T cells in the organs of ecCpG adjuvanted mice was also manifested in absolute cell numbers (Figure 2C). These data indicate that one mechanism by which adjuvant ecCpG drives memory CD8 T cell response is to increase the number of antigen-specific CD8 T cells whereby ecCpG is superior to scCpG adjuvant in doing so. Intriguingly, ecCpG and scCpG drove specific CD8 T cells both in the lymphoid organs and at a distant site from where the antigen was re-introduced.

### Epicutaneous CpG ODN Adjuvant Promotes Strong Memory CD8 T Cell Response at the Site of Influenza A Viral Infection

**Figure 3:**
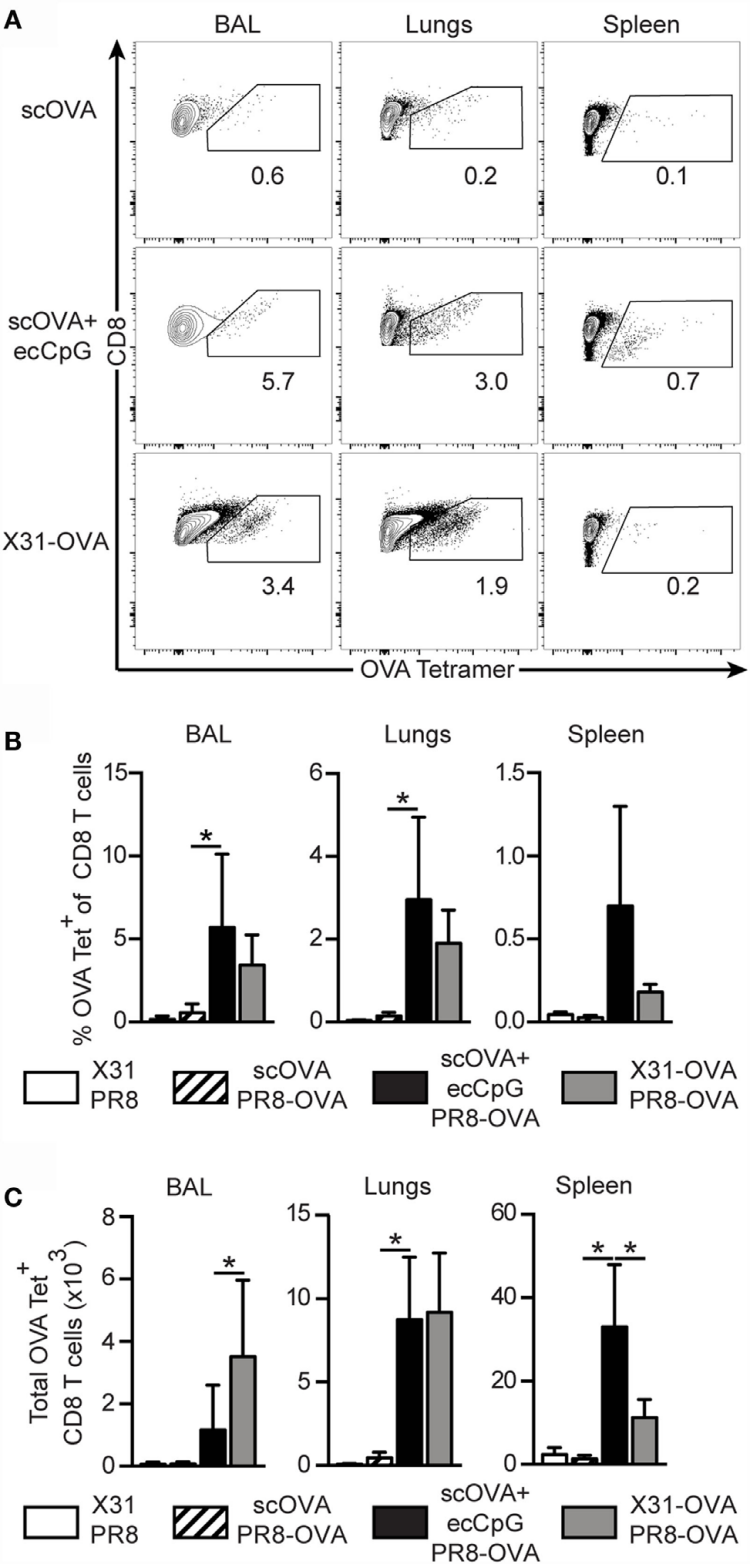
**Epicutaneous CpG ODN induces memory CTL responses equivalent to primary influenza viral infection**. C57BL/6 mice immunized with scOVA ± ecCpG ODN or intranasally infected with X31-OVA virus were challenged with PR8-OVA virus 30 days post-immunization. The BAL, lungs, and spleen were collected 3 days post-challenge for flow cytometric analysis and OVA-specific CD8 T cells were enumerated. **(A)** Representative flow cytometric plots of the B220^−^CD8^+^ population, gating for OVA tetramer^+^ CD8^+^ double-positive cells. **(B)** The percentage and **(C)** total number of OVA-specific cells among CD8^+^ T cells in the BAL, lungs, and spleen, including the negative controls of mice immunized with X31 and challenged with PR8. Bar graphs are showing the mean ± SD from a representative experiment from three independent experiments (*n* = 3–6). Statistical significance of all four groups was analyzed by one-way ANOVA test with a Tukey’s multiple comparison *post hoc* analysis. *p < 0.05.

Given the increase in antigen-specific CD8 memory T cells after immunization with ecCpG adjuvant and reintroduction of the antigen, we next investigated the generation of memory T cells upon re-challenge of immunized mice with live IAV. PR8 IAV or virus expressing an OVA epitope recognized by CD8 T cells (PR8-OVA) was used to challenge the mice 30 days post-immunization to examine antigen-specific responses. The less virulent IAV, X31, and its derivative that express the CD8 T cell epitope of OVA (X31-OVA) were used to infect the mice. Mice were then challenged with PR8 and PR8-OVA as negative and positive controls, respectively, for memory OVA-specific CD8 T cell generation. Three days after challenge, we examined the response in the lungs, spleen, and BAL, which samples the epithelial lining of the pulmonary airways. Significantly higher percentages of OVA-specific CD8 T cells were detected in ecCpG adjuvanted mice compared to mice immunized with antigen alone in all the compartments analyzed (Figures 3A,B). The memory response in the lungs was comparable between mice immunized with scOVA + ecCpG and mice infected with X31-OVA upon challenging with PR8-OVA. Increased numbers of OVA-specific CD8 T cells were detected in the lungs and spleen of mice immunized with scOVA + ecCpG compared to scOVA alone. Importantly, equivalent numbers of OVA-specific CD8 T cells were detected in the lungs of scOVA + ecCpG immunized mice compared to X31-OVA infected mice (Figure 3C). These results confirm the ability of ecCpG adjuvant to induce memory antigen-specific CD8 T cell response in the lungs. Using ecCpG as an adjuvant can effectively enhance memory CD8 T cell response at the sites of IAV infection, including the airways and the lungs, which were as strong as that generated by primary IAV infection.

### Epicutaneous CpG Adjuvant Stimulates a Strong Humoral Response

**Figure 4:**
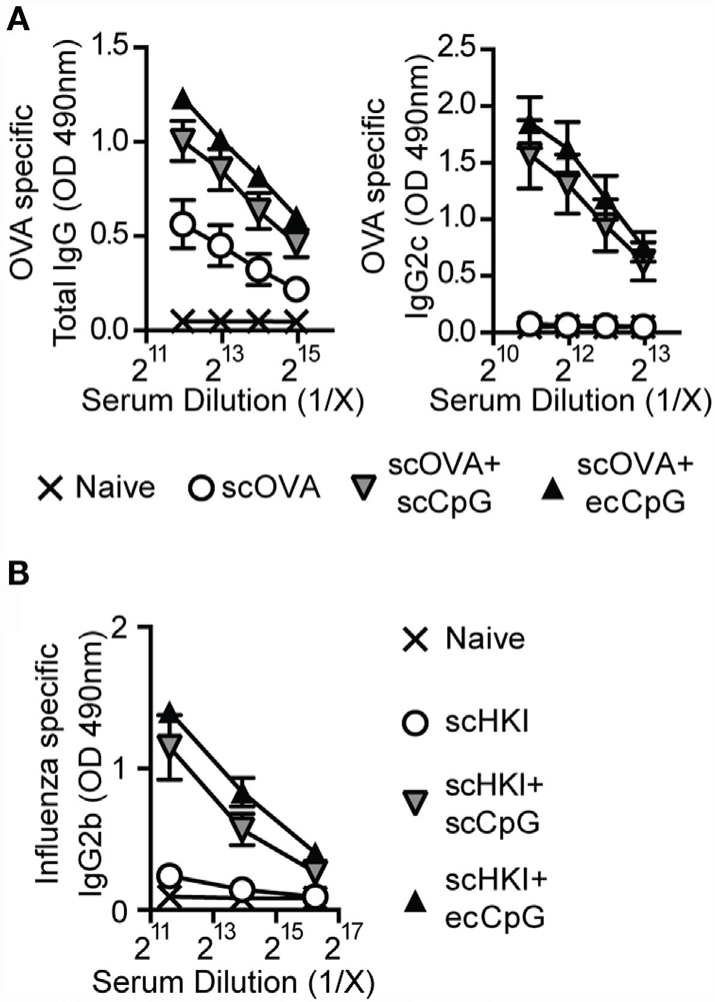
**Epicutaneous CpG ODN enhances antibody responses to influenza virus**. C57BL/6 mice immunized with scOVA ± ecCpG or scCpG ODN, or subcutaneously with heat-killed IAV (scHKI) ± ecCpG or scCpG ODN. Sera were collected 30 days post-immunization and serial diluted. Relative levels of **(A)** OVA-specific total IgG, IgG2c, and **(B)** IgG2b antibodies in serum from the different groups of mice were measured by ELISA. Line graphs show the mean ± SD of antibody levels from two independent experiments (*n* = 6).

Antibody responses remain a standard method to evaluate vaccines and are essential in long-term protective immunity. Mice were immunized using a prime-boost schedule with scOVA, scOVA + scCpG, or scOVA + ecCpG. The relative titers of OVA-specific total IgG and IgG2c antibodies in the serum were measured 30 days post-immunization (Figure 4A). Independent of the route of administration, CpG ODN adjuvant increased the amount of total IgG and IgG2c relative to immunization without adjuvant. To mimic influenza vaccines, we also measured relative antibody titers in mice subcutaneously immunized with heat-killed PR8 (scHKI). As speculated, increased IgG2b antibodies were detected with CpG ODN adjuvant regardless of the route of delivery compared to antigen alone (Figure 4B).

### Epicutaneous CpG Adjuvant Stimulates a Strong Th1 Response from CD4 T Cells

**Figure 5:**
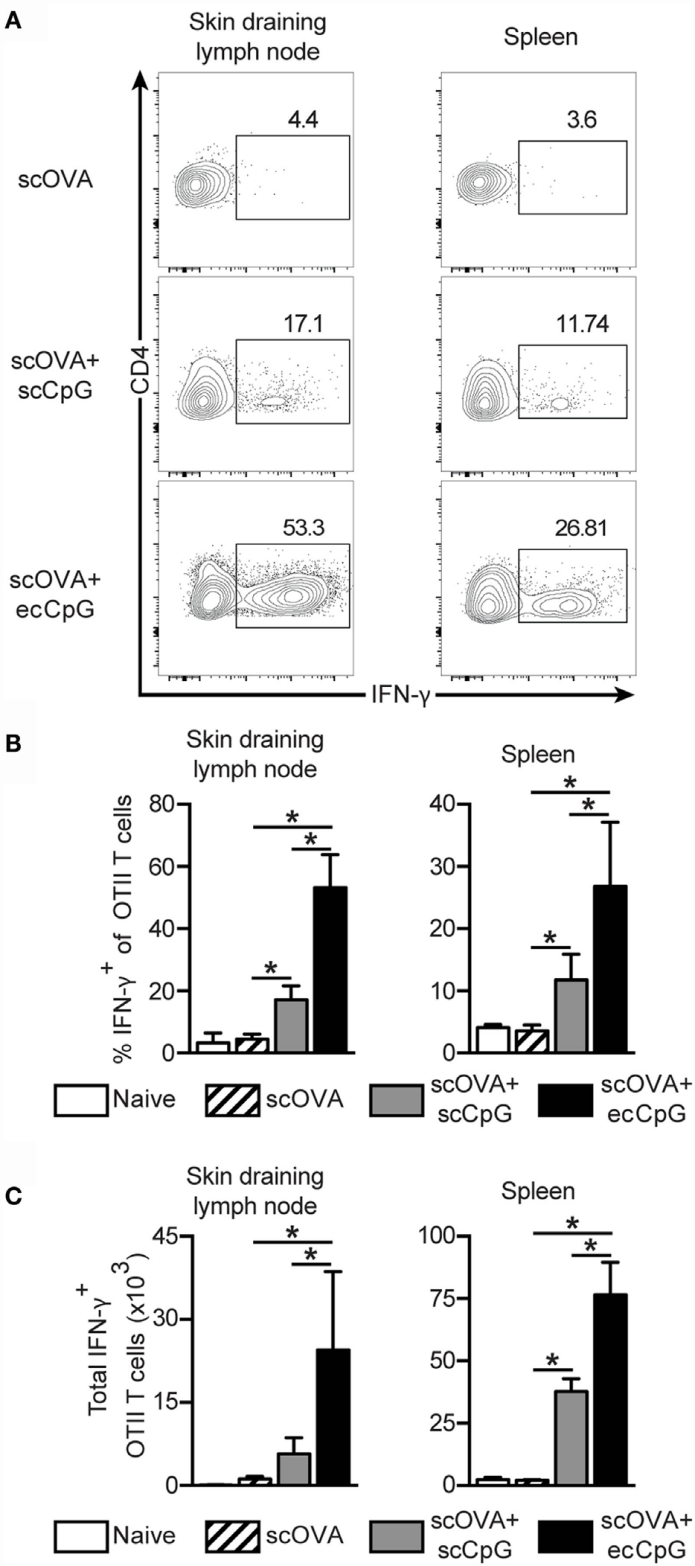
**Epicutaneous CpG ODN enhances Th1 responses**. C57BL/6 mice adoptively transferred with CD4+ Thy1.1+ OT-II T cells were immunized with scOVA ± ecCpG or scCpG ODN. Skin draining lymph nodes and spleen were collected 3 days after and cells were restimulated with PMA and ionomycin *ex vivo* for 4 h. Cells producing IFN-γ were detected by intracellular IFN-γ staining and flow cytometric analysis. **(A)** Representative flow cytometric plots of CD4^+^ and Thy1.1^+^ cells, gating for CD4 IFN-γ double-positive cells is shown. **(B)** The percentage of IFN-γ producing cells among CD4^+^ Thy1.1^+^ OT-II T cells in the skin draining lymph nodes and spleen. **(C)** Total number of IFN-γ producing CD4^+^ Thy1.1^+^ OT-II T cells in the skin draining lymph nodes and spleen. Bar graphs are showing the mean ± SD from a representative experiment from three independent experiments (*n* = 3–4). Statistical significance of all four groups was analyzed by one-way ANOVA test with a Tukey’s multiple comparison *post hoc* analysis. *p < 0.05.

CD4 T cells not only provide help to B cell clonal expansion and antibody production (45), they also play important roles in the development and maintenance of functional CD8 memory T cells (46, 47). The CD4 Th1 cell subset is characterized by the production of IFN-γ. There is evidence that IFN-γ can act directly on CD8 T cells to increase their number during acute viral infection (48). The OT-II adoptive transfer system was used to examine the effect of ecCpG adjuvant on CD4 T cells. Mice were first adoptively transferred with CD4 T cells from OT-II T cell receptor transgenic mice carrying the Thy1.1 congenic allele, then immunized with scOVA alone, scOVA + scCpG, or scOVA + ecCpG. After 3 days, cells were isolated from the spleen and skin draining lymph nodes and restimulated with PMA and ionomycin *ex vivo*. Although there was no difference in the number of Thy1.1 OT-II cells, intracellular cytokine staining showed a fourfold increase in the proportion of IFN-γ-producing Thy1.1 OT-II cells in the lymphoid organs with scOVA + scCpG immunization compared to scOVA alone (Figures 5A,B). When mice were immunized with scOVA + ecCpG, we observed an increase in IFN-γ-producing Thy1.1 OT-II cells in the skin draining lymph nodes and the spleen compared to scOVA + scCpG immunization (Figures 5A,B). Similarly, the total number of IFN-γ-producing Thy1.1 OT-II cells were also significantly increased in the skin draining lymph nodes and the spleen with ecCpG adjuvant (Figure 5C). These results suggest that ecCpG is superior to scCpG adjuvant in stimulating IFN-γ production from antigen-specific CD4 T cells.

### Epicutaneous CpG Adjuvant Modulates Homing Molecules on CD4 T Cells

**Figure 6:**
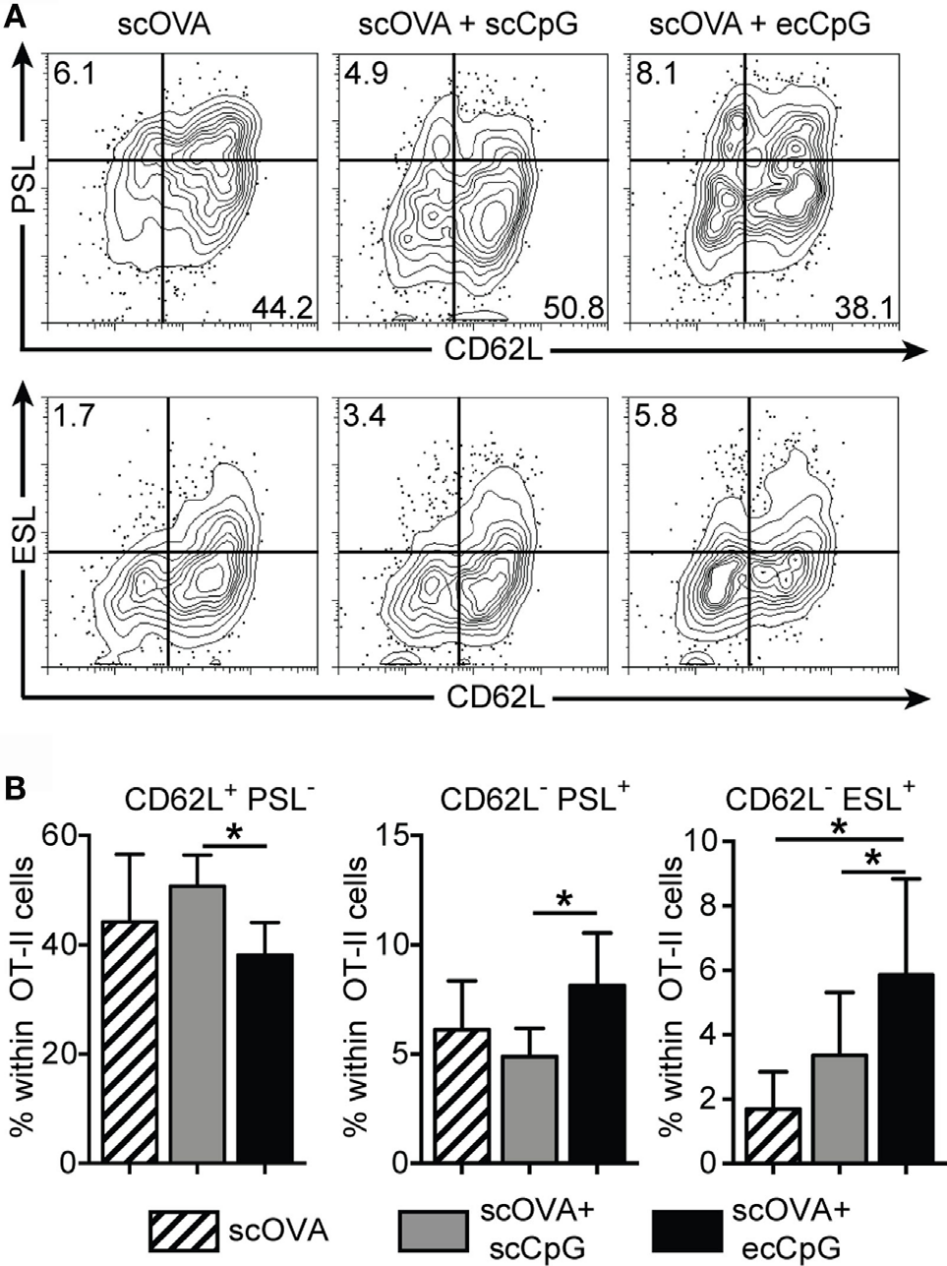
**Epicutaneous CpG ODN enhances P- and E-selectin ligand expression in CD4 antigen-specific T cells**. C57BL/6 mice adoptively transferred with CD4^+^ Thy1.1^+^ OT-II T cells were immunized with scOVA ± ecCpG ODN. Skin draining lymph nodes were collected for flow cytometric analysis 3 days post-immunization. **(A)** Representative flow cytometric plots of CD4 Thy1.1 double-positive cells showing selectin ligand expression. **(B)** The percentage of single-positive cells for CD62L, PSL, or ESL among CD4^+^ Thy1.1^+^ cells in the skin draining lymph nodes. Bar graphs show the mean ± SD of 4 individual experiments, *n* = 9. Statistical significance of all three groups was analyzed by one-way ANOVA test with a Tukey’s multiple comparison *post hoc* analysis. *p < 0.05.

Immunization with ecCpG adjuvant drove memory CD8 T cell response in the lungs upon intranasal influenza viral infection (Figure 3). P-selectin ligand (PSL) and E-selectin ligand (ESL) are molecules required for the transmigration of T cells from the endothelium to blood into lymphoid organs (49). Moreover, P-selectin binds only to antigen-specific T cells in draining lymph nodes after immunization (50). It has been shown that independent of the route of herpes simplex viral infection, CD8 central memory T cells expressed substantial levels of the ESL and PSL during both the effector and memory phase of the response. By contrast, epicutaneous infection led to significantly higher expression of tissue-homing molecules in CD4 helper T cells (51). CD4 T cells affect the outcome of CD8 responses, by enhancing clonal expansion, facilitating the entry of effectors into inflamed tissues, and promoting the survival of memory cells, all of which are preceded by the tuning of antigen-presenting cells in lymph nodes by the CD4 cells (52). We hypothesized that ecCpG adjuvant with subcutaneous protein antigen upregulates the expression of tissue-homing molecules on antigen-specific T cells. Using a CD4 OT-II transgenic T cell adoptive transfer approach, we investigated the expression of tissue-homing molecules on antigen-specific cells after immunization. Mice were adoptively transferred with Thy1.1 OT-II transgenic T cells and then immunized the next day with scOVA, scOVA + scCpG, or scOVA + ecCpG. The skin draining lymph nodes were analyzed 3 days post-immunization. Epicutaneous delivery of CpG ODN adjuvant increased the proportion of OT-II cells expressing PSL and ESL compared to scCpG delivery (Figure 6). By contrast, the proportion of the OT-II cells expressing L-selectin (CD62L) was decreased. These data are consistent with the hypothesis that ecCpG adjuvant promotes expression of the homing molecules of PSL and ESL. In addition, the ecCpG adjuvant reduces expression of lymph node homing molecule CD62L.

## Discussion

The route of vaccination and the adjuvant applied governs the immune responses to vaccination with protein antigens. Thus, developing a universal IAV vaccine involves the investigation of the optimal delivery of the vaccine. Cutaneous immunization is able to generate protective resident memory T cell populations at distal mucosal sites in various infection and vaccination settings (53). In addition, the TLR9 agonist CpG ODN preferentially induces a strong CD4 Th1 and CD8 immune response (54). During the 2009 H1N1 pandemic, prior elevated frequencies of influenza-specific CD8 T cells correlated with protection against symptomatic disease (14). The induction of a strong, CTL response may, thus, confer broader and more persistent protection from IAV infection as the epitopes recognized by CD8 T cells are more conserved.

In this study, we investigated whether the combination of subcutaneous protein immunization with ecCpG adjuvant could induce protective memory T cell responses to IAV. Our immunization and challenge system allowed us to specifically examine protection driven by memory CD8 T cells. This was accomplished since we immunized and boosted with the whole ovalbumin protein plus CpG ODN as the adjuvant. However, challenge of the immunized mice was done with the PR8-OVA virus, with only the MHC-I restricted ovalbumin epitope, SIINFEKL, as the common antigen between immunization and challenge. We showed that CpG ODN as an adjuvant generated a protective CD8 T cell response to IAV challenge, and that route of delivery was critical for optimal response and protection. While CpG ODN delivered subcutaneously with antigen was able to generate a memory CD8 T cell response, only epicutaneous delivery generated a robust CD8 T cell response that conferred protection against IAV infection. This suggests that epicutaneous application of CpG ODN adjuvant is superior to subcutaneous administration.

After an infection, memory T cells form distinct populations of tissue resident and migratory cells. There are three major subsets of memory T cells: central memory, effector memory, and tissue resident memory T cells (55, 56). Central memory T cells are CD127^+^ CD62L^+^, have a higher proliferative potential than naive cells, and preferentially reside in lymphoid organs. Effector memory T cells are CD127^+^ CD62L^−^, have a faster effector response, and preferentially reside in non-lymphoid (stromal) tissues. Unlike central memory T cells, Trm permanently reside in their home tissue and do not recirculate into the blood (31). Trm are conditioned by the tissue environment in which they arise (57). Transferring Trm cells to a naïve animal will result in nearly all of them returning to the imprinted tissue (58). Interestingly, memory cells generated in the skin are able to home to multiple tissues (59). The mechanism by which the skin can program T cells to home to other sites has not been determined. By contrast, central memory T cells patrol lymph nodes and tissues for antigen but do not permanently reside in the tissues or have any tissue-specific conditioning (58). While both of these populations confer protection against reinfection, Trm cells tend to be more effective (56). Their location proximal to the site of infection enables them to respond rapidly to reinfection and they are able to efficiently recruit help from the innate immune system and the circulating memory T cell populations. Our results demonstrated that immunization with ovalbumin and ecCpG adjuvant was effective at generating a protective secondary CD8 T cell response to PR8-OVA infection in the lungs and the BAL fluid. Although the nature of the memory CD8 population generated upon immunization with scOVA + ecCpG is still undefined, these cells responded equally well to IAV reinfection as memory CD8 T cells generated by a primary IAV infection and are consistent with the presence of a Trm population.

The peripheral response to re-challenge with PR8-OVA was quantitatively different between mice vaccinated with scOVA + ecCpG and infected with X31-OVA. The secondary T cell response in X31-OVA primed mice was confined to the BAL and the lungs. This is typical of a memory T cell response to IAV. However, in mice vaccinated with scOVA + ecCpG, we detected a strong CD8 T cell response in the spleen, unlike mice that were initially infected with IAV. This indicated that skin vaccination might have induced a larger circulating memory CD8 T cell population than primary IAV infection, leading to an enhanced secondary response. The implications of an enhanced peripheral CD8 T cell response to IAV re-challenge include enhanced vaccine efficacy; an outcome that will need to be clinically tested.

While memory CD8 T cell responses have the potential to provide cross-serotype immunity against IAV, they cannot completely prevent reinfection like protective humoral immunity (1). In addition to generating a protective CD8 T cell response, CpG ODN adjuvant enhanced the production of antigen-specific IgG antibodies, independent of the route of administration. When we examined the CD4 T cell population, we found that ecCpG was superior to scCpG ODN adjuvant in generating the IFN-γ-producing Th1 cells. Although the role of CD4 T cells in protection from IAV infection is still debated, they are recognized to contribute to the clearance of IAV (60). Taken together, it remains beneficial to elicit a humoral response against influenza strains in addition to the induction of CD8 T cell memory.

The development of the adaptive immune response is influenced by innate immune cells and their context of activation. The skin is spatially segregated into layers with different stromal and immune cell types in each layer. Thus, there is both functional and spatial separation of the immune response between cells. The difference in response between mice treated with scCpG versus ecCpG adjuvant demonstrates the importance of separation of function in immune priming. It is important to dissect the effects of treatments to different layers in modulating the local immune response. In this setting, stimulation of the epidermis was critical for the generation of protective CD8 T cell memory. ecCpG enabled the cross-presentation of soluble protein antigen-specific CD8 T cells administered either intramuscularly or subcutaneously (42). The main cell types in the epidermis that express TLR9 and recognize CpG ODN are keratinocytes and Langerhans cells (LCs). Langerhans cells activated by CpG ODN are able to migrate to draining lymph nodes and cross-present antigen to CD8 T cells (42). Stimulation of keratinocytes through TLR9 triggers the production and release of cytokines and chemokines, such as TNF-α, IL-1α, and type I interferons (61). Furthermore, cytokines and chemokines released by keratinocytes after stimulation by CpG ODN enhance the ability of LCs to cross-present and activate CD8 T cells (62). It is likely that CpG ODN stimulation of keratinocytes, LCs, or both was essential for priming of CD8 T cells in the skin to a memory population that protected against IAV challenge in the lungs.

It is important for vaccines to generate both memory B and T cells to provide long-term protection. Higher frequencies of antigen-specific memory T cells correlate with enhanced protection (63, 64). Due to their higher activation state, memory T cells respond to pathogen encountered in a faster and stronger manner in subsequent exposures (65). CD4 T cells help direct and tune CD8 T cell responses. We investigated the effect of immunization with ecCpG compared to scCpG adjuvant in modulating selectin expression that would correlate with the homing potential of antigen-specific CD4 T cells. Results from our study supports the hypothesis that ecCpG adjuvant promotes the expression of tissue-homing molecules on memory T cells, including PSL and ESL that was not detected with subcutaneous delivery of the adjuvant. By contrast, the expression of lymph node-retaining molecule CD62L was decreased with epicutaneous delivery of CpG ODN adjuvant but not with subcutaneous delivery. This effector memory T cells pattern of adhesion molecules may allow memory T cells to migrate peripheral tissues and to mount a strong response against IAV to provide the first line of defense at the site of infection.

Vaccination is one of the most effective public health interventions in use today. Transcutaneous immunization is a promising delivery route for the development of new and improved vaccines and adjuvants. We have demonstrated that standard subcutaneous protein vaccination with ecCpG ODN adjuvant generated a strong memory CD8 T cell response that conferred protection from IAV challenge in the lungs. The combination of delivery route and adjuvant was important for an effective memory CD8 T cell response and may be applicable to other vaccine settings. Results from this study can guide future endeavors in understanding the mechanism of ecCpG ODN adjuvant enhancement of the efficacy of IAV and other vaccines to induce both protective T cell and humoral response.

## Author Contributions

The study was conceived and designed by WC, AP, NA and JD. WC and AP preformed experiments. WC, AP, and JL analyzed the data. Manuscript was written by WC, AP, and JL and edited by NA and JD.

## Conflict of Interest Statement

The authors declare that the research was conducted in the absence of any commercial or financial relationships that could be construed as a potential conflict of interest.

## Abbreviations

BAL, broncheoalveolar lavage; CpG ODN, CpG oligo deoxynucleotide; ec, epicutaneous; ESL, E-selectin ligand; HAU, hemagglutinin units; IAV, influenza A virus; PSL, P-selectin ligand; sc, subcutaneous; Trm, tissue-resident memory T cells.
